# Raza, degeneración y eugenesia en América Latina

**DOI:** 10.1590/S0104-59702025000100028

**Published:** 2025-06-16

**Authors:** Óscar Gallo

**Affiliations:** i Departamento de Historia/Facultad de Ciencias Sociales y Humanas/Universidad de Antioquia.Medellin – Antioquia – Colombia ofernando.gallo@udea.edu.co

De acuerdo con las normas de publicación de reseñas, estas deben tener un título diferente de la obra reseñada. Mi título no tan diferente quiere remarcar una idea: “La teoría de la degeneración operó como condición histórica de posibilidad para el surgimiento, difusión y aceptación de las teorías eugenésicas defendidas en las primeras décadas del siglo xx” (Olaya Peláez, González Bernaldo de Quirós, Márquez Valderrama, 2024, p.211). En efecto, la eugenesia precedida por la constatación de la degeneración y la tradición de los debates contemporáneos sobre raza, población, pueblo, país o nacionalidad (véase Solodkow, 2022, p.80) posibilitó el “clima eugenésico” de América Latina. Este es el horizonte de la provocadora obra colectiva en la que participaron 23 investigadores con una profusa trayectoria en historia de la ciencia, historia de la medicina e historia sociocultural de la enfermedad.

La obra reseñada es más que la reiteración de “*The hour of eugenics:” race, gender, and nation in Latin America*, de Nancy Stepan (1991), aunque sea citada en una parte importante de los artículos que configuran la obra y varios de los autores coincidan con la tesis latina de la historiadora. Con otras palabras, al indagar por los modos operatorios más difusos, más diversos, menos sensacionalistas o los espacios sociales inéditos de la eugenesia, como observa Iván Olaya junto con Paul-André Rosental y Luc Berlivet, los autores pueden más que “afirmar o negar la existencia de una eugenesia latinoamericana …, destacar la disponibilidad de una caja de herramientas eugenésicas que, a la luz de la metodología situada, permite ver las particularidades locales de sus aplicaciones” (p.4).

En efecto, en las tres últimas décadas se publicaron más de una centena de investigaciones directa o indirectamente relacionadas con la cuestión, pero ninguna, como *Raza, eugenesia y políticas públicas en América Latina, 1900-1950*, logra juntar las particularidades locales de las aplicaciones de la eugenesia en países tan diversos como Argentina, Brasil, Chile, Colombia, Cuba, Ecuador y México. Con frecuencia, la historiografía sobre la salud, la enfermedad y la medicina en América Latina se centra principalmente en los casos de Brasil y Argentina. Como crítica, cabe señalar que la obra excluye – lamentablemente – las realidades del Caribe, Centroamérica y de países como Perú y Bolivia. En ese sentido, para solo mencionar un caso, los análisis de Paulo Drinot (2017) sobre la defensa de la raza peruana hubieran encontrado una concordancia armoniosa con el análisis del “problema del indio” ecuatoriano realizada por Emmanuelle Sinardet o las ficciones nacionales mexicanas de Pablo Yankelevich. Aunque una obra no puede abarcar la totalidad de la región, pues se volvería desmesurada, es como mínimo intrigante pensar las realidades de la única república negra del continente, Haití, o los imaginarios raciales de su vecino República Dominicana, analizados por Milagros Ricourt (2022).

Ahora bien, ¿cómo se usó esa caja de herramientas eugenésicas en América Latina? Según los autores, en políticas públicas orientadas a la selección de la migración y en las acciones dirigidas a la protección materno infantil. Pero también destacan los expertos convocados el combate pedagógico a las enfermedades venéreas, el destaque de las biotipologías y la cultura física con su énfasis en el motor humano, el sesgo normativo de la maternidad, el racismo de la psiquiatría cubana, las restricciones sanitarias frente a la migración del Medio Oriente, el impulso dado a la migración sueca, las redes tecno-científicas que promocionaron esa especie de panamericanismo eugenésico, las particularidades del caso uruguayo o las políticas sociales orientadas a la incorporación de los indígenas a la nación. De lo anterior se puede concluir que más que las necedades de una ciudad letrada que compartía ideas en torno a la raza, el “clima eugenésico”, en un contexto de positivismo y medicalización de la política, era el “prolegómeno de la acción” y legitimaba con argumentos biológicos los “proyectos sociales y la promulgación de leyes públicas que buscaban valorizar el capital humano de la nación” (Olaya Peláez, González Bernaldo de Quirós, Márquez Valderrama, 2024, p.473). Desde este punto cabe recordar a Diego Armus, citado por Eric Carter (2023, p.40), cuando afirma que “la eugenesia se situaba en la intersección de la biología y la política y era … más bien una manifestación racionalizada de la necesidad y el deseo de controlar y dominar a una población … Era en gran medida una forma de hablar de los problemas sociales en términos biológicos”.

Para concluir, si tuviera a los autores enfrente, estas serían algunas de mis preguntas. En primer lugar, ¿qué otros modos operatorios más difusos, más diversos, menos sensacionalistas o inéditos se pueden encontrar además de la infancia y la migración? En segundo lugar, dado que los humanos somos raras veces conscientes de nuestro presente, pasado y futuro, ¿la práctica es suficiente para encuadrar el fenómeno? ¿O, para decirlo más claramente, se puede afirmar que los contemporáneos eran conscientes de estar actuando en consonancia con el proyecto eugenésico? Valdría la pena recordar al historiador Eric Carter (2023) cuando afirma que los actores históricos de principios del siglo tenían enfrente límites indefinidos y conexiones complejas con otras epistemes, por ejemplo, la higiene, la medicina del trabajo, la salud pública, la medicina rural, la racionalidad productiva, el socialismo y el anarquismo. En tercer lugar, ¿qué lugar ocupó la religión en la comprensión de lo humano y el progreso? Aunque en el libro no omite completamente la alusión a la religión, queda la sensación de que los Estados eran bastante laicos en sus políticas. Finalmente, todos los casos incluidos en la obra refuerzan la hipótesis latina de la eugenesia ¿Qué se puede ampliar de los otros casos que no coinciden con la perspectiva eugenésica en la época? Con todo, las preguntas anteriores solo pretenden insinuar algunos de los problemas, interrogantes y perspectivas planteados en la obra. Sin duda, se trata de un trabajo con análisis originales sobre un fenómeno de relevancia regional que, en mi opinión, es fundamental para comprender el papel intervencionista del Estado en diversas esferas de la vida.
